# Activation of SIK1 by phanginin A regulates skeletal muscle glucose uptake by phosphorylating HADC4/5/7 and enhancing GLUT4 expression and translocation

**DOI:** 10.1007/s13659-025-00504-z

**Published:** 2025-04-07

**Authors:** Yu Shi, Xing-de Wu, Yanli Liu, Yu Shen, Hui Qu, Qin-Shi Zhao, Ying Leng, Suling Huang

**Affiliations:** 1 State Key Laboratory of Drug Research, Shanghai Institute of Materia Medica, Chinese Academy of Sciences, Shanghai, 201203 China; 2 University of Chinese Academy of Sciences, Beijing, 100049 China; 3 State Key Laboratory of Phytochemistry and Plant Resources in West China, Kunming Institute of Botany, Chinese Academy of Sciences, Kunming, 650201 China; 4 Key Laboratory of Ethnic Medicine Resource Chemistry, State Ethnic Affairs Commission & Ministry of Education, Yunnan Minzu University, Kunming, 650500 Yunnan China

**Keywords:** SIK1, Phanginin A, Class IIa HDACs, Skeletal muscle, GLUT4, Glucose uptake

## Abstract

Salt-inducible kinase 1 (SIK1) participates in various physiological processes, yet its involvement in regulating skeletal muscle glucose uptake remains unclear. Previously, we showed that phanginin A, a natural compound isolated from *Caesalpinia sappan Linn*, activated SIK1 to suppress gluconeogenesis in hepatocytes. Here, we aimed to elucidate the effects of SIK1 on skeletal muscle glucose uptake by using phanginin A. The C2C12 myotubes were incubated with phanginin A and then glucose uptake, mRNA levels, membrane GLUT4 content, phosphorylation levels of proteins in SIK1/HDACs and Akt/AS160 signaling pathways were determined. Phanginin A significantly promoted glucose uptake, while the pan-SIK inhibitor or knocking down SIK1 expression abolished the promotion. Further exploration showed that phanginin A enhanced GLUT4 mRNA levels by increasing histone deacetylase (HDAC) 4/5 phosphorylation and MEF2a mRNA and protein level, and knocking down SIK1 blocked these effects. Additionally, phanginin A induced HDAC7 phosphorylation, upregulated the junction plakoglobin (JUP) expression and Akt/AS160 phosphorylation. Knocking down JUP or SIK1 both attenuated the phanginin A-induced Akt/AS160 signaling and glucose uptake, suggesting that activation of SIK1 by phanginin A inactivated HDAC7 to increase JUP expression and Akt/AS160 phosphorylation, led to upregulation of GLUT4 translocation and glucose uptake. *In vivo* study showed that phanginin A increased phosphorylation levels of SIK1, HDAC4/5/7, Akt/AS160, and gene expression of MEF2a, GLUT4 and JUP, accompanied by elevated membrane GLUT4 and glycogen content in gastrocnemius muscle of C57BL/6 J mice, indicating enhanced glucose utilization. These findings reveal a novel mechanism that SIK1 activation by phanginin A stimulates skeletal muscle glucose uptake through phosphorylating HADC4/5/7 and the subsequent enhancement of GLUT4 expression and translocation.

**Graphical abstract:**

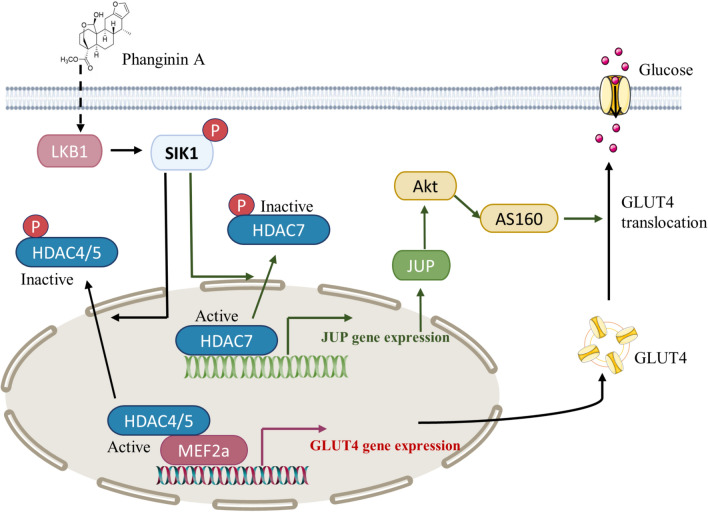

## Introduction

Skeletal muscle is the largest metabolic organ in human body, plays crucial roles in glucose homeostasis, accounting for approximately 80% of glucose conversion and 75% of glycogen storage in postprandial state [1]. Glucose transporter 4 (GLUT4) serves as a primary transporter for glucose in muscle cells, with its translocation from cytoplasm to cytoplasmic membrane determines the rate of glucose uptake in skeletal muscle [2]. This process is pivotal for modulating skeletal muscle glucose transport and muscle glycogen content, as evidenced in diabetic mice [3]. Insulin triggers GLUT4 translocation by activating phosphatidylinositol 3-kinase (PI3K)/protein kinase B (PKB, Akt) signaling [4]. Additionally, various factors such as exercise-caused contraction, AMP-activated protein kinase (AMPK), nitric oxide (NO), reactive oxygen species (ROS), and calcium can regulate GLUT4 translocation [5, 6]. The expression of GLUT4 gene is primary controlled by transcription factors including myocyte enhancer factor 2 (MEF2) and glucose enhancer factor (GEF) [7]. Studies showed that class IIa histone deacetylases (HDACs) bind to MEF2 to inhibit its transcription activity, and exercise or other signals might regulate the phosphorylation level of HDACs to relieve the inhibition [8, 9]. Deciphering the key signaling components that regulate GLUT4 expression and trafficking is still vital for understanding skeletal muscle glucose utilization.

Salt-induced kinase 1 (SIK1) is a serine/threonine protein kinase, belonging to the AMPK-related kinases (AMPKRK) family, and is involved in energy response-related physiological processes [10, 11]. It serves as a crucial regulator of various cellular activities, with HDACs and CREB-regulated transcriptional co-activators (CRTCs) among its well-proven substrates [12, 13]. Knocking down SIK1 in myoblast impaired the process from myoblast differentiating to myotubes [14]. Mechanistic investigations revealed that SIK1 promotes cell growth and differentiation in skeletal muscle by phosphorylating HDAC5, leading to its export from the nucleus and subsequent relief inhibition on MEF2 activity [15]. Despite these insights, whether SIK1 modulates GLUT4 expression and glucose uptake in fully differentiated myotubes remains unexplored, warranting further investigation into this aspect.

Junction plakoglobin (JUP), also referred as γ-catenin, serves as a cytosolic component of adherens junctions and desmosomes, playing a crucial role in cell signaling transduction [16]. In lung cancer cells, JUP transcription is negatively regulated by HDAC7, a member of class IIa HDACs closely related to the known SIK substrates HDAC4 and HDAC5 [17]. Additionally, studies by Negoita and colleagues have shown that inhibiting SIK2, an isoform of SIK1, reduces JUP expression and impairs the insulin signaling pathway in adipocytes [18]. More recently, JUP expression has been detected in skeletal muscle, where it interacts with p85 of PI3K to augment PI3K/Akt signaling and glucose uptake [19]. Given the demonstrated metabolic effects of SIK1 mediated by class IIa HDACs, it merits exploration to investigate whether SIK1 influences JUP expression, thereby potentially regulate glucose uptake in skeletal muscle.

*Caesalpinia sappan Linn.* is a shrubby tree well distributed in Yunnan of China. Its heartwood is used as a natural medicine in the treatment of gynecological diseases and injuries. Phanginin A is a diterpenoid compound isolated from seeds of *C. sappan*., with few pharmacological activities been reported. In our prior study, we discovered that phanginin A can inhibit hepatic gluconeogenesis by promoting SIK1 phosphorylation [20]. In this investigation, we found that phanginin A enhanced glucose uptake in C2C12 myotubes dependent on SIK1 activation. Further exploration revealed a novel mechanism by which SIK1 activation, induced by phanginin A, enhances skeletal muscle glucose uptake through two complementary pathways: increasing GLUT4 expression and promoting GLUT4 translocation, mediated by the inactivation of HDAC4/5/7.

## Results

### Phanginin A enhanced glucose uptake in C2C12 myotubes

**Fig. 1:**
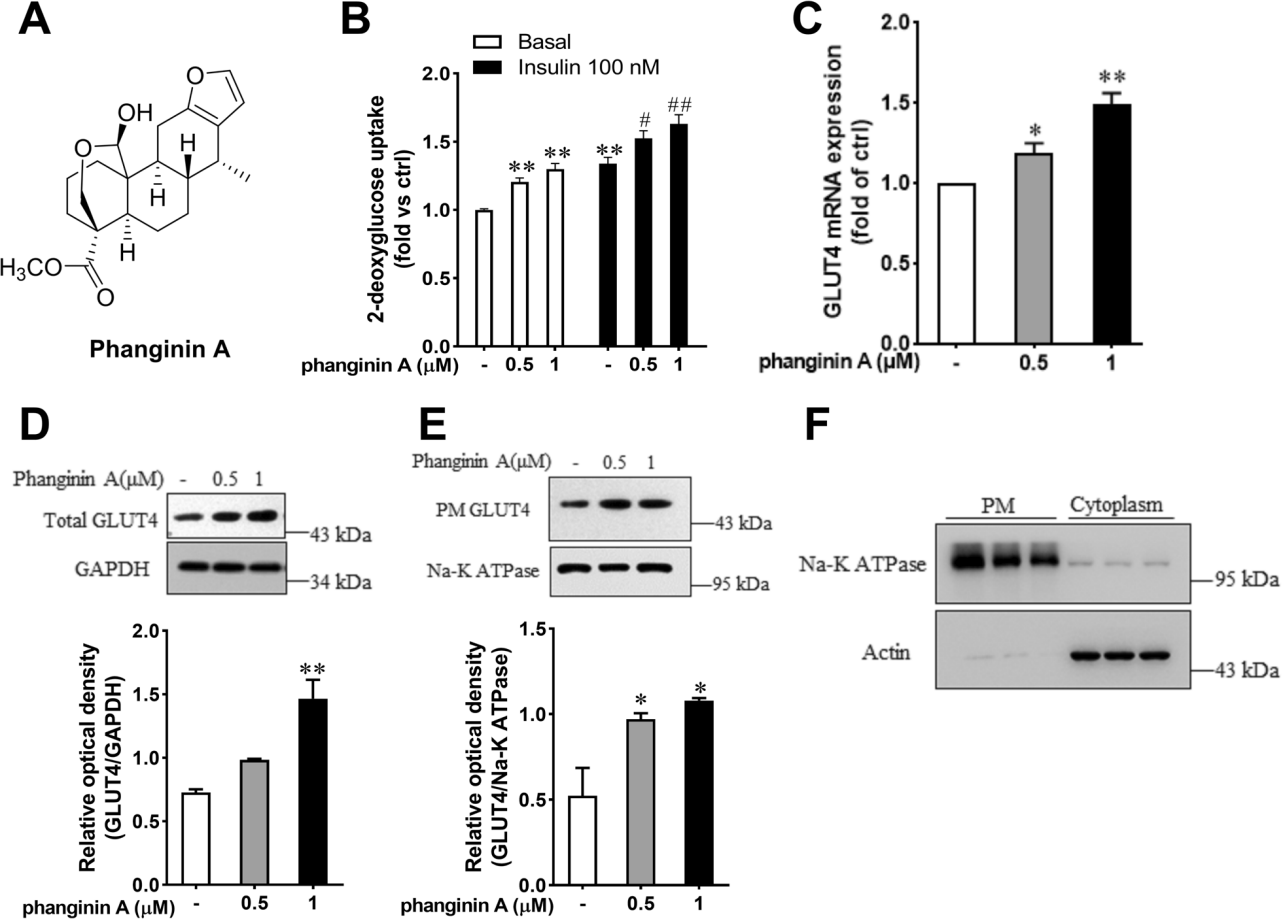
Phanginin A enhanced glucose uptake in C2C12 myotubes. **A** Chemical structure of phanginin A. **B** Effect of phanginin A on glucose uptake in C2C12 myotubes under basal and insulin-induced conditions. **C** Effect of phanginin A on GLUT4 mRNA expression. **D** Effect of phanginin A on the total GLUT4 protein of C2C12 myotubes homogenates. **E** GLUT4 translocation, as determined by GLUT4 protein abundance in plasma membrane protein fractions. **F** Purity of the isolated plasma membrane protein fractions (used to determine GLUT4 translocation in **E**), as determined by immunoblot analysis of Na + /K + -ATPase subunit α1 (plasma membrane protein marker) in plasma membrane homogenates (PM) and actin (cytosoplasmic protein marker) in cytoplasm. Data represent mean ± SEM (n = 3). ^∗^P < 0.05 and ^∗∗^P < 0.01 vs controls under basal conditions. ^#^P < 0.05 and ^##^P < 0.01 vs control under insulin-induced conditions

Phanginin A (Fig. 1A) significantly enhanced glucose uptake of C2C12 myotubes under both basal and insulin-stimulated conditions. Incubation with 1 μM phanginin A caused a comparable increase in glucose uptake to that observed with 100 nmol/L insulin (Fig. 1B). Both mRNA and total protein levels of GLUT4 were augmented by phanginin A (Fig. 1C, D). The translocation of GLUT4 to the cell membrane is a critical factor influencing glucose uptake in skeletal muscle [21]. To further investigate whether phanginin A also affects GLUT4 translocation, we employed a complementary approach to measure GLUT4 protein abundance in plasma membrane fractions [22]. The plasma membrane fractions and cytoplasmic fractions were isolated using a commercial membrane extraction kit. Na^+^/K^+^-ATPase subunit α1 was used as a plasma membrane protein marker and actin as a cytoplasmic protein marker (Fig. 1F). As showed in Fig. 1E, there was a notable increase in GLUT4 protein abundance in the plasma membrane after phanginin A treatment, indicating that phanginin A promotes GLUT4 translocation in C2C12 myotubes.

### Phanginin A stimulated glucose uptake in C2C12 myotubes by activating SIK1

**Fig. 2:**
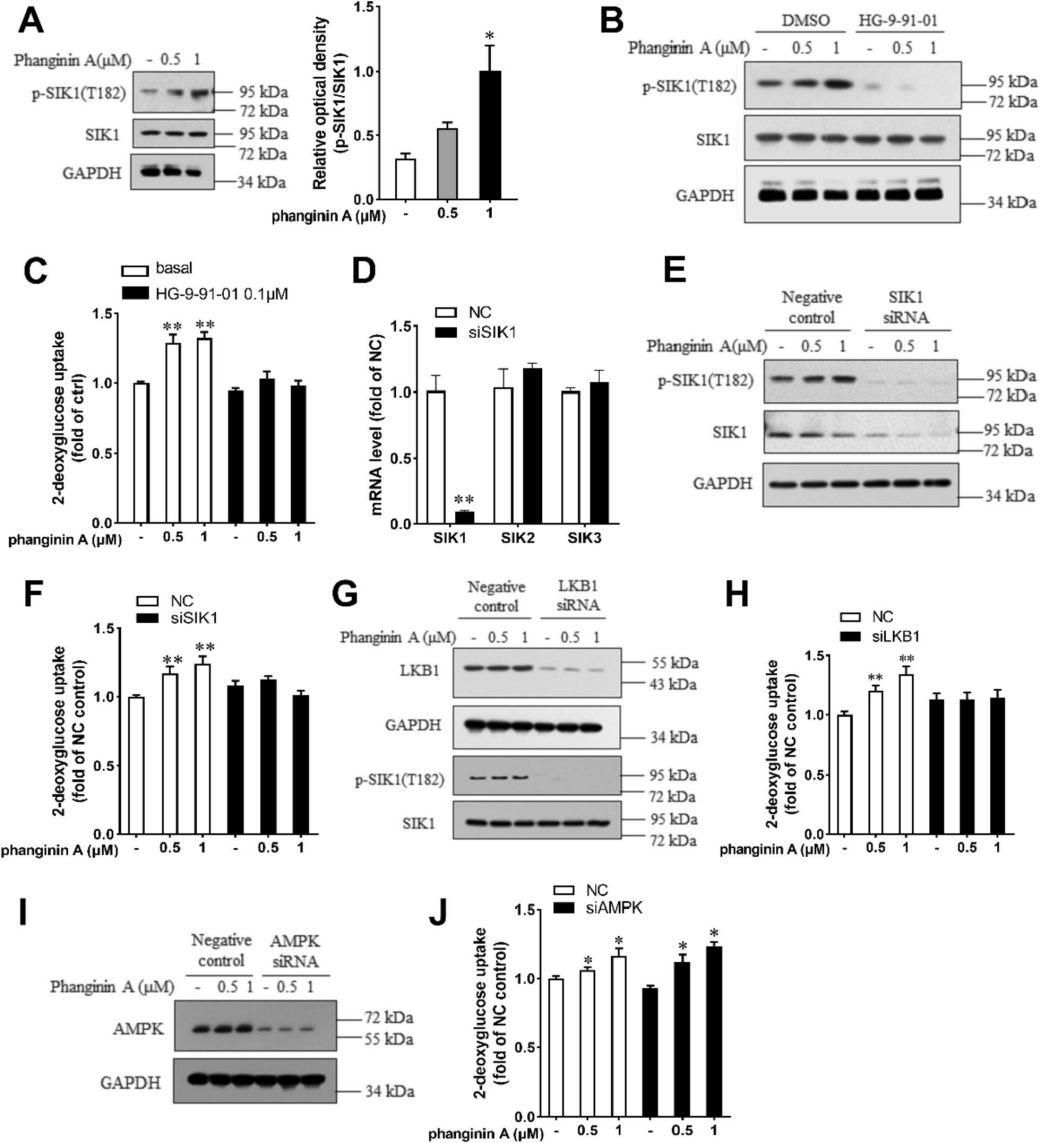
Phanginin A stimulated glucose uptake in C2C12 myotubes by activating SIK1. **A** C2C12 myotubes were treated with phanginin A for 24 h, followed by western blot analysis to assess the phosphorylation level of SIK1 at Thr182. **B**, **C** C2C12 myotubes were pre-treated with or without 0.1 μM of HG-9-91-01 for 30 min, then co-treated with phanginin A. Subsequently, the SIK1 phosphorylation level (**B**) and glucose uptake (**C**) were evaluated. **D**–**F** C2C12 myotubes were pre-treated with SIK1 siRNA for 48 h, followed by phanginin A treatment, and then the mRNA levels of SIK1-3 (**D**), SIK1 phosphorylation, and protein levels (**E**), along with glucose uptake (**F**) were measured. **G**, **H** C2C12 myotubes were pre-treated with LKB1 siRNA for 48 h, followed by phanginin A treatment. Subsequently, LKB1 protein, SIK1 phosphorylation levels (**G**) and glucose uptake (**H**) were determined. **I**, **J** C2C12 myotubes were pre-treated with AMPK siRNA for 48 h, followed by phanginin A treatment. Then the AMPK protein levels (**I**) and glucose uptake (**J**) were assessed. Data are presented as mean ± SEM (n = 3), ^∗^P < 0.05 and ^∗∗^P < 0.01 vs control

Given that phanginin A promoted phosphorylation of the SIK1 at Thr182 in liver, which represented SIK1’s activated state, we investigated its effect on SIK1 phosphorylation in skeletal muscle. As expected, Phanginin A notably increased SIK1 phosphorylation at Thr182 in C2C12 myotubes (Fig. 2A). To ascertain whether phanginin A-stimulated glucose uptake relied on SIK1 activation, we utilized the pan-SIK inhibitor HG-9-91-01 to suppress SIK activity. Treatment with HG-9-91-01 completely abrogated both phanginin A-stimulated SIK1 phosphorylation (Fig. 2B) and glucose uptake (Fig. 2C). Following siRNA interference, SIK1 expression was substantially reduced compared to the negative control group, with no discernible changes in SIK2 and SIK3 expression (Fig. 2D). As expected, phanginin A failed to stimulate SIK1 phosphorylation and glucose uptake upon SIK1 silencing (Fig. 1E, F), underscoring the dependence of phanginin A-induced glucose uptake on SIK1. Considering LKB1 as an upstream kinase of SIK1, phosphorylating and activating it [23], we knocked down LKB1 expression via RNA interference in C2C12 myotubes (Fig. 2G). The phanginin A-induced SIK1 phosphorylation and the promotion of glucose uptake were abolished upon knocking down LKB1 (Fig. 2G, H). Although AMPK serves as another substrate of LKB1 and involved in regulating glucose uptake in skeletal muscle, knocking down AMPK did not impede the phanginin A-stimulated glucose uptake (Fig. 2I, J). Collectively, these findings indicate that phanginin A enhances glucose uptake in skeletal muscle through a SIK1-dependent mechanism.

### Activation of SIK1 by phanginin A increased GLUT4 expression via phosphorylating class IIa HDACs in C2C12 myotubes

**Fig. 3:**
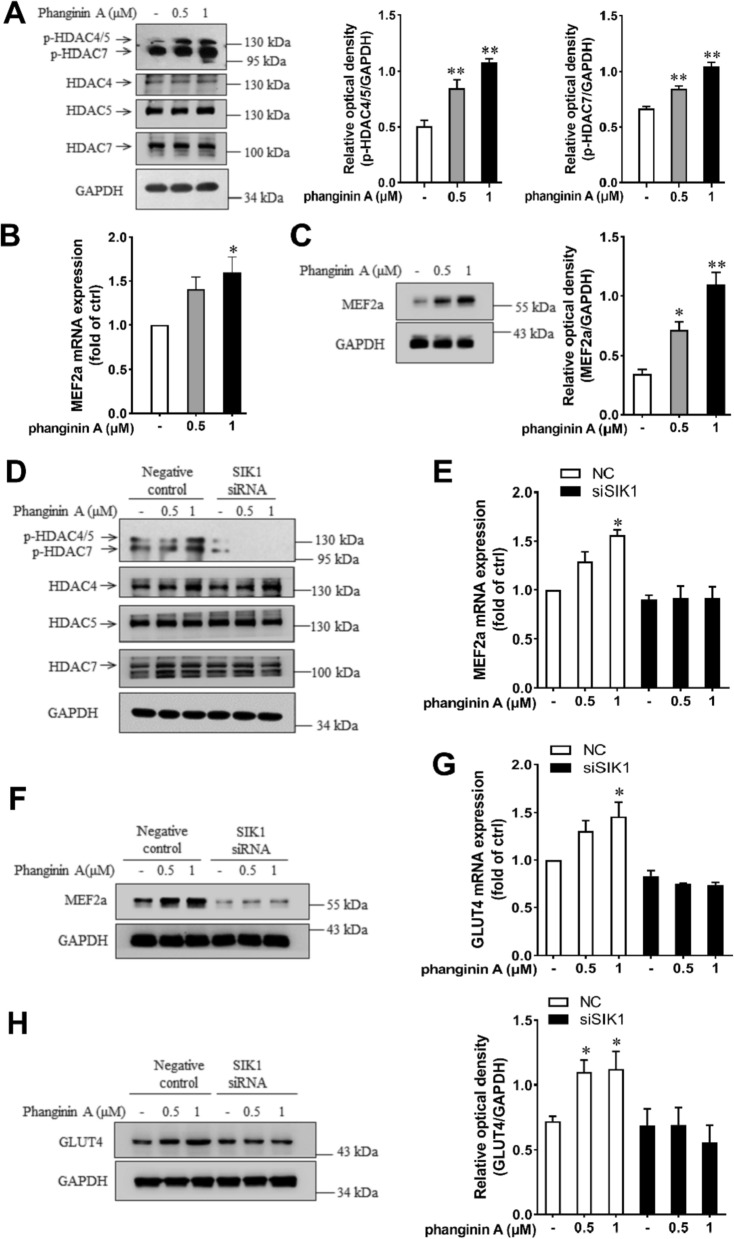
Activation of SIK1 by phanginin A enhanced GLUT4 expression through phosphorylating HDAC4/5 in C2C12 myotubes. **A** Phosphorylation levels of HDAC4/5/7 after phanginin A treatment. **B**, **C** Effect of phanginin A on MEF2a mRNA level (**B**) and protein level (**C**). **D**–**H** C2C12 myotubes were pre-treated with SIK1 siRNA for 48 h, then exposed to phanginin A for another 24 h. Then the phosphorylation levels and corresponding total protein levels of HDAC4/5/7 (**D**), MEF2a mRNA levels (**E**), MEF2a protein levels (**F**), GLUT4 mRNA levels (**G**), and GLUT4 protein levels (**H**) were assessed. Data presented as the mean ± SEM (n = 3), ^∗^P < 0.05 and ^∗∗^P < 0.01 vs control

Previous reports showed that SIK1 regulates myogenic differentiation through phosphorylating Class IIa HDACs to relieve suppression on MEF2 [15, 24]. Here, phanginin A treatment caused a significant increase in the phosphorylation levels of HDAC4/5/7 (Fig. 3A). Given the established role of MEF2a in regulating GLUT4 expression [9], we investigated whether SIK1 and MEF2a were involved in phanginin A-stimulated GLUT4 gene expression. As anticipated, phanginin A markedly elevated the mRNA level (Fig. 3B) and protein level (Fig. 3C) of MEF2a. Upon SIK1 siRNA interference, phanginin A failed to enhance the phosphorylation of HDAC4/5 and HDAC7 (Fig. 3D). Additionally, the upregulation of mRNA and protein levels of MEF2a (Fig. 3E, F) and GLUT4 (Fig. 3G, H) were also abolished. These findings suggest that activation of SIK1 by phanginin A increases GLUT4 expression through phosphorylation of Class IIa HDACs in C2C12 myotubes.

### Activation of SIK1 by phanginin A stimulated glucose uptake in C2C12 myotubes through upregulating JUP expression and Akt/AS160 signaling pathway

**Fig. 4:**
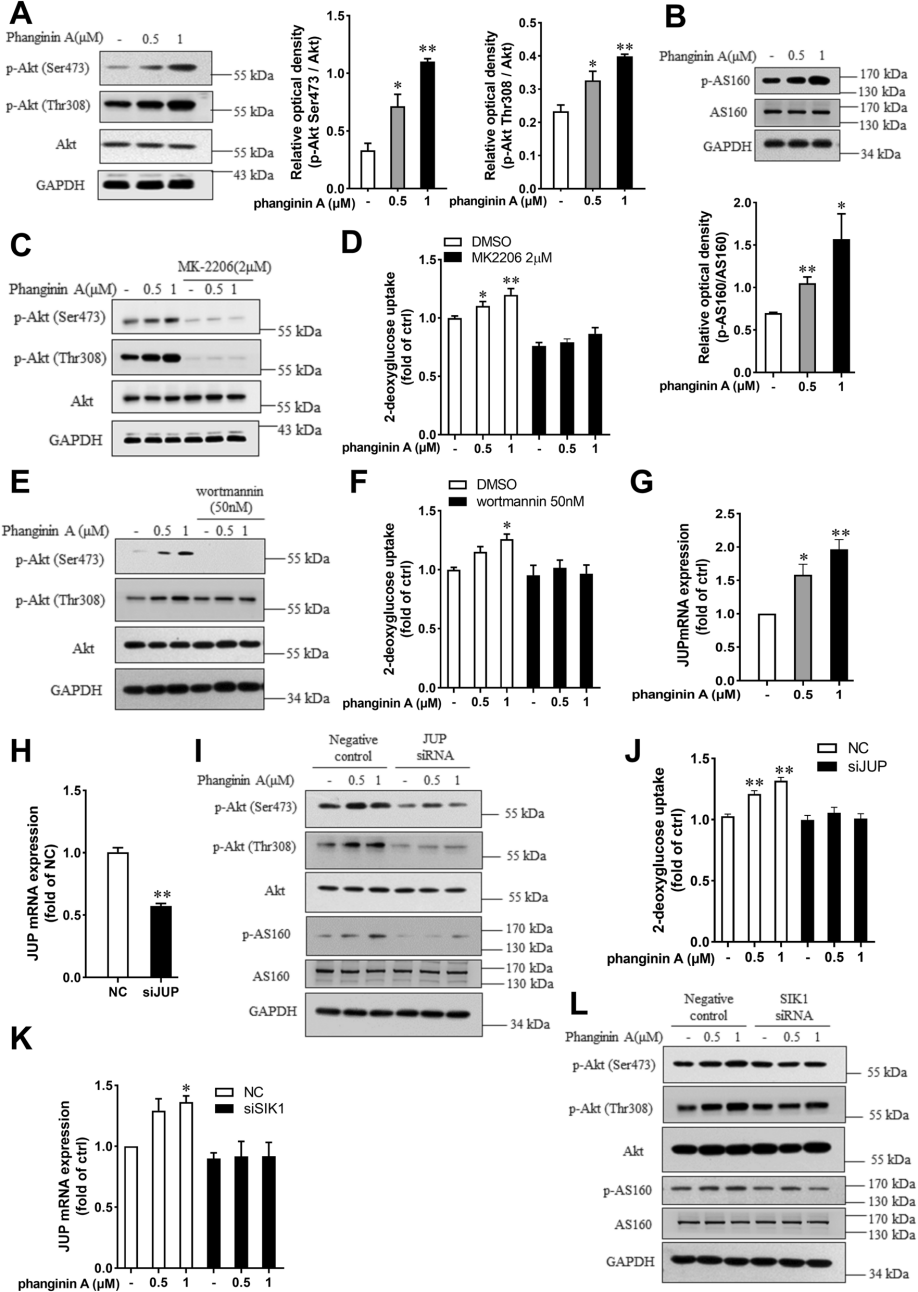
Activation of SIK1 by phanginin A stimulated glucose uptake in C2C12 myotubes through upregulating JUP expression and Akt/AS160 signaling pathway. **A**, **B** Phosphorylation levels of Akt and AS160 in C2C12 myotubes were assessed by western blot after treatment with 0.5 or 1 μM of phanginin A. **C**, **D** C2C12 myotubes were pretreated with or without 2 μM of MK2206 for 30 min and then cotreated with phanginin A, followed by measurement of Akt and AS160 phosphorylation (**C**) and glucose uptake (**D**). **E**, **F** Akt phosphorylation level (**E**) and glucose uptake (**F**) were assessed in C2C12 myotubes pretreated with or without 50 nM of wortmannin for 30 min and then cotreated with phanginin A. **G** Relative mRNA level of JUP in C2C12 myotubes was assessed after treatment with 0.5 or 1 μM of phanginin A. **H** JUP mRNA level in C2C12 myotubes was assessed following siRNA-mediated JUP knockdown. **I**, **J** Western blot analysis of phosphorylation levels of Akt and AS160 (**I**) and glucose uptake (**J**) after JUP knockdown (**H**). **K**, **L** The mRNA levels of JUP (**K**) and phosphorylation levels of Akt and AS160 (**L**) were assessed in C2C12 myotubes following siRNA-mediated SIK1 knockdown. Data are shown as mean ± SEM (n = 3), ^∗^P < 0.05 and ^∗∗^P < 0.01 vs control

The Akt/AS160 (Akt substrate of 160 kDa) pathway is crucial for regulating GLUT4 translocation [25, 26]. We wondered whether Akt/AS160 signaling was involved in the phanginin A-increased the GLUT4 protein abundance in plasma membrane of C2C12 myotubes. After phanginin A treatment, the Akt phosphorylation at Ser473 and Thr308, as well as the AS160 phosphorylation at Thr642 were significantly increased in C2C12 myotubes (Fig. 4A, B). Pretreatment of myotubes with Akt inhibitor MK2206 fully blocked the phanginin A-stimulated Akt phosphorylation and glucose uptake (Fig. 4C, D). The PI3K inhibitor wortmannin also abrogated the phanginin A-induced Akt phosphorylation (Fig. 4E) and glucose uptake (Fig. 4F), indicating phanginin A stimulates glucose uptake dependent on PI3K/Akt signaling pathway. We further explored the manner how phanginin A upregulates Akt phosphorylation. JUP was taken into consideration for it was reported negatively regulated by HDAC7, one of the class IIa HDACs [17], and HDAC7 was significantly phosphorylated and inactivated after phanginin A treatment (Fig. 3A). Phanginin A upregulated the gene expression of JUP, with a twofold increase after 1 μM incubation (Fig. 4G). To ascertain JUP’s role as a key mediator between SIK1 and Akt signaling, we knocked down JUP expression using siRNA in C2C12 myotubes, reducing JUP expression to 50% of the negative control (Fig. 4H). Subsequent assays revealed that downregulation of JUP blocked the phanginin A-induced phosphorylation of Akt and AS160, as well as glucose uptake (Fig. 4I, J). Furthermore, knocking down SIK1 abrogated phanginin A’s effect on the upregulation of JUP mRNA expression (Fig. 4K) and phosphorylation of Akt and AS160 (Fig. 4L), indicating that activation of SIK1 by phanginin A upregulates JUP expression to enhance the Akt/AS160 signaling pathway and thus stimulates glucose uptake.

### Phanginin A treatment increased phosphorylation of SIK1 and HDAC4/5/7, leading to upregulation of GLUT4 expression and Akt/AS160 pathway in skeletal muscle of C57BL/6 J mice

**Fig. 5:**
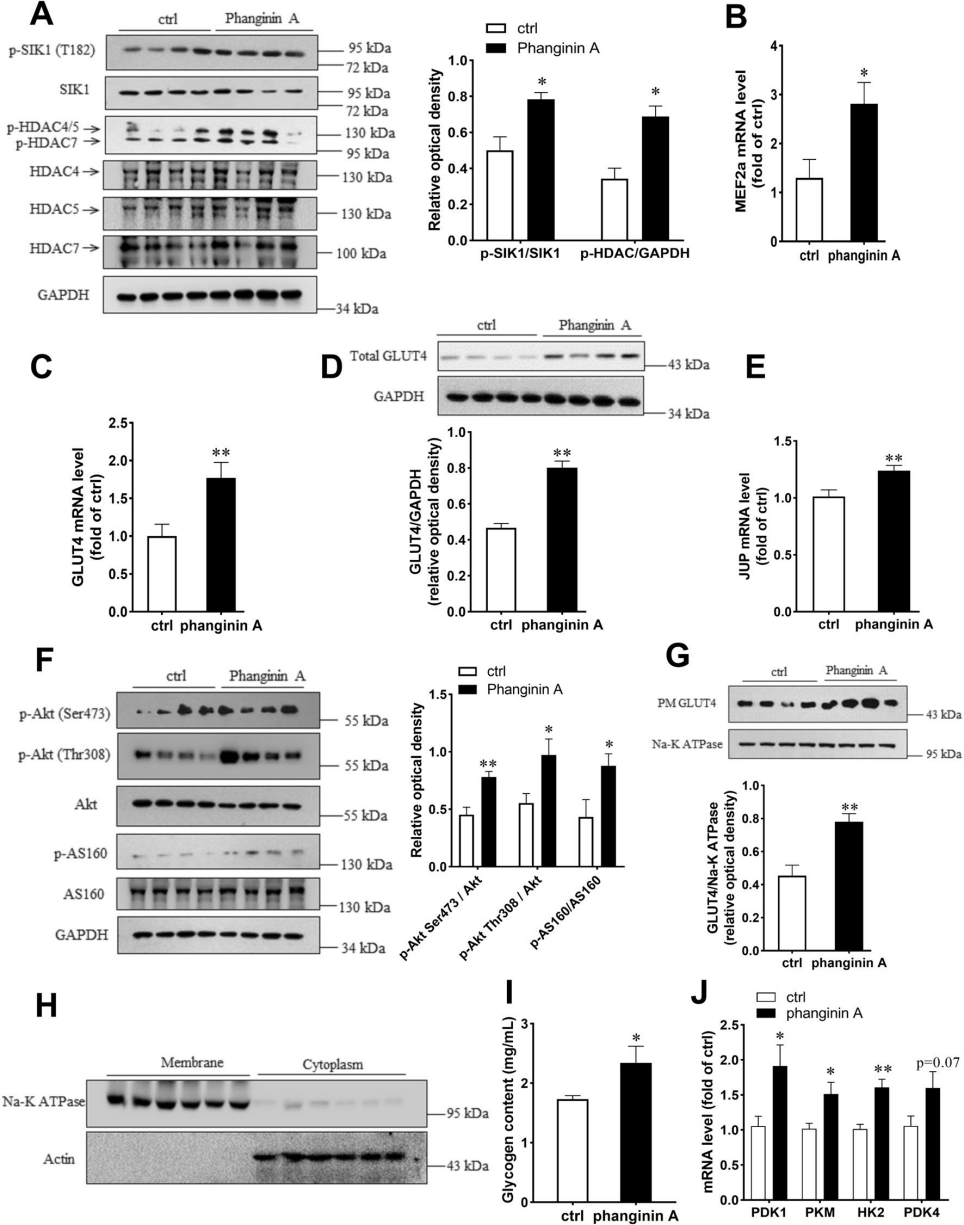
Activation of SIK1 by phanginin A modulated glucose metabolism capacity in gastrocnemius muscles of C57BL/6 J mice. Mice were treated as described in the Methods section. **A** Western blot analysis of the phosphorylation levels and corresponding total protein levels of SIK1 and HDAC4/5/7 in the gastrocnemius muscle. **B**–**D** Relative mRNA levels of MEF2a (**B**) and GLUT4 (**C**), along with the protein level of GLUT4 (**D**) in gastrocnemius muscle were evaluated. **E**, **F** Relative mRNA level of JUP, and the phosphorylation levels and corresponding total protein levels of Akt and AS160 in gastrocnemius muscle were also measured. **G** Western blot analysis illustrated GLUT4 content in the plasma membrane of gastrocnemius muscle. (**H**) Purity of the isolated membrane protein fractions (used to determine GLUT4 in **G**) of gastrocnemius muscle, as determined by immunoblot analysis of Na ^+^ /K ^+^ -ATPase subunit α1 in membrane homogenates and actin in cytoplasm. (**I**) Quantification of glycogen content in the gastrocnemius muscle. (**J**) Relative mRNA level of PDK1, PDK4, PKM and HK2 in gastrocnemius muscle. Values represent the mean ± SEM (n = 8), *P < 0.05 and **P < 0.01 vs control

To investigate the effects of phanginin A on skeletal muscle glucose metabolism *in vivo*, phanginin A (100 mg/kg) was administered once daily to C57BL/6 J mice for 16 days. As shown in Fig. 5A, phanginin A significantly elevated the phosphorylation level of SIK1 at T182 in gastrocnemius muscle, and the phosphorylation level of HDAC4/5/7 was also significantly increased. The mRNA levels of MEF2a and GLUT4 were markedly augmented by phanginin A in the gastrocnemius muscle (Fig. 5B, C), with a corresponding upregulation in GLUT4 protein levels (Fig. 5D). Notably, phanginin A also significantly increased JUP mRNA level (Fig. 5E) and enhanced the phosphorylation levels of Akt at Ser473 and Thr308, and AS160 at Thr642 by 63, 76 and 103%, respectively (Fig. 5F). Given that glucose uptake relies on GLUT4 translocation, and it serves as the initial rate-limiting step in skeletal muscle glucose utilization and glycogen synthesis, we assessed membrane GLUT4 protein content, and key genes involved in glycolysis. The membrane protein fractions of gastrocnemius muscle homogenates were isolated (Fig. 5H) and used to determine GLUT4 abundance. The membrane GLUT4 abundance was significantly higher in phanginin A-treated group, suggesting enhanced GLUT4 translocation (Fig. 5G). The glycogen content in gastrocnemius muscle was significantly increased by phanginin A (Fig. 5I). The mRNA level of key glycolytic genes including pyruvate dehydrogenase kinase isozyme 1 (PDK1), pyruvate kinase (PKM), hexokinase 2 (HK2), and pyruvate dehydrogenase kinase isozyme 4 (PDK4) were also significantly upregulated (Fig. 5J), suggesting increased glucose utilization.

## Discussion

SIK1 attracts increasing attention for its’ multiple roles in the regulation of energy metabolism and cell growth [27]. While previous research in skeletal muscle has predominantly focused on its involvement in myogenic differentiation [28], its precise mechanism in skeletal muscle glucose uptake remains unclear. Our prior investigation revealed that phanginin A activates hepatic SIK1 via LKB1, thereby inhibiting hepatic gluconeogenesis [20]. In our current study, we found that phanginin A also induces SIK1 phosphorylation in skeletal muscles, and the phanginin A-enhanced skeletal muscle glucose uptake was dependent on SIK1 activation. Our findings reveal a novel mechanism wherein SIK1 activation by phanginin A inactivates Class IIa HDACs, leading two distinct yet complementary effects: the upregulation of GLUT4 expression and the facilitation of GLUT4 translocation to the plasma membrane, collectively enhancing glucose uptake.

Phosphorylation of SIK1 at Thr182 is crucial for motivating its kinase catalytic activity [24]. Phanginin A promoted the phosphorylation of SIK1 at Thr182 and enhanced glucose uptake in C2C12 myotubes. Pan-SIK inhibitor HG-9-91-01 entirely abolished the phanginin A-stimulation on glucose uptake, suggesting SIK activity was indispensable. Since HG-9-91-01 also inhibits the activity of SIK2 and SIK3, two isoforms of SIK1 [29], SIK1 was knocked down by RNA interference to further specify the association of phanginin A-increased SIK1 activation and glucose uptake. As expected, interference of SIK1 expression fully reversed phanginin A-stimulated glucose uptake. Other SIK isoforms can be exclude, for the expression of SIK2 and SIK3 were not influenced by SIK1 interference. Additionally, we investigated the role of LKB1, an upstream regulator of SIK1 phosphorylation. Knocking down LKB1 expression prevented the phanginin A-induced elevation of SIK1 phosphorylation and subsequent glucose uptake. Despite AMPK being another downstream protein of LKB1, and known for promoting glucose uptake in skeletal muscle [30, 31], knocking down AMPK did not affect the phanginin A-stimulated glucose uptake. Therefore, the stimulation of glucose uptake by phanginin A was dependent on the enhancement of SIK1 phosphorylation through LKB1.

Class IIa HDACs, including HDAC4, 5, 7 and 9, are known substrates of SIKs [13]. It is well recognized that SIK1 phosphorylates HDAC4/5 to exert its pleiotropic effects on metabolism [32], while HDAC7 was reported to be regulated by SIK2 in adipocytes [18]. Here, we found that activation of SIK1 by phanginin A induced the phosphorylation of HDAC4/5/7 in C2C12 myotubes. HDAC4/5 negatively regulated the activity of transcription factor MEF2, a crucial regulator of muscle development by binding to it [33]. Phosphorylation of HDAC4/5 would lead to their extrusion from the nucleus, thus permitting MEF2 to activate target genes’ transcription [15]. GLUT4, the most crucial glucose transporter in skeletal muscle, is one of the target genes controlled by MEF2 [21]. However, whether SIK1 regulates skeletal muscle GLUT4 expression hasn’t been reported. Our findings showed that phanginin A increased the phosphorylation of HDAC4/5, as well as the mRNA and protein levels of MEF2a and GLUT4. Importantly, these effects were abolished in SIK1 knocked down C2C12 myotubes, suggesting that activation of SIK1 could upregulate GLUT4 expression via HDAC4/5-MEF2a axis in myotubes.

The translocation of GLUT4 is primarily regulated by the classic PI3K/Akt/AS160 signaling pathway [34]. Here, GLUT4 translocation, phosphorylation of Akt and AS160 were significantly enhanced by phanginin A. PI3K inhibitor wortmannin reversed the activation of Akt and glucose uptake enhanced by phanginin A. Similar results were found with Akt inhibitor MK-2206, indicating that PI3K/Akt is indispensable in the stimulation of glucose uptake. It has been reported that JUP, which is negatively regulated by HDAC7, can interact with the PI3K/Akt signaling pathway [19]. HDAC7 is a member of Class IIa HDACs [17, 32], and phanginin A enhanced the phosphorylation level of HDAC7. Knocking down of SIK1 resulted in a decreased phosphorylation of HDAC7, suggesting HDAC7 is also a substrate of SIK1. Thus we hypothesized that JUP might be the mediator between SIK1 and Akt signaling. Indeed, our results revealed that phanginin A upregulates the expression of JUP, and siRNA-mediated knockdown of JUP abolished the phanginin A-stimulated activation of Akt/AS160 signaling and glucose uptake, confirming JUP’s role as a mediator. Furthermore, the upregulation of JUP and Akt/AS160 signaling induced by phanginin A was completely abolished upon SIK1 interference in C2C12 myotubes, further supporting the dependence of these effects on SIK1. Therefore, activation of SIK1 by phanginin A could inactivate HDAC7 to increase JUP expression and thus enhanced Akt/AS160 pathway to promote glucose uptake in skeletal muscle.

Given that glucose uptake is the most important rate-limiting step of skeletal muscle glucose utilization [4], we further investigated the effects of phanginin A on skeletal muscle glucose metabolism of C57BL/6 J mice. Consistent with the in vitro findings, a 16-day treatment with phanginin A markedly increased the phosphorylation levels of SIK1 and HDAC4/5/7 in the gastrocnemius muscle. This enhancement was accompanied by elevated mRNA levels of MEF2a and GLUT4, along with an increase in GLUT4 protein abundance. Furthermore, there was an upregulation in JUP gene expression, activation of the Akt/AS160 signaling pathway, and an increase membrane GLUT4, indicating enhanced glucose transport in skeletal muscle. After transported into cells, the glucose would be further synthesized into muscle glycogen for storage or glycolysis for energy production [35]. Here, phanginin A treatment increased the glycogen content and the expression of key genes involved in glycolysis of gastrocnemius muscle, suggesting it promoted the further utilization of glucose within skeletal muscle. In summary, our in vivo data support the notion that administration of phanginin A increased SIK1 activation, threrby facilitating the uptake and utilization of glucose through inactivation of HDAC4/5/7. These findings suggest that phanginin A may hold promise for enhancing skeletal muscle glucose metabolism and its associated beneficial effects.

## Conclusion

**Fig. 6:**
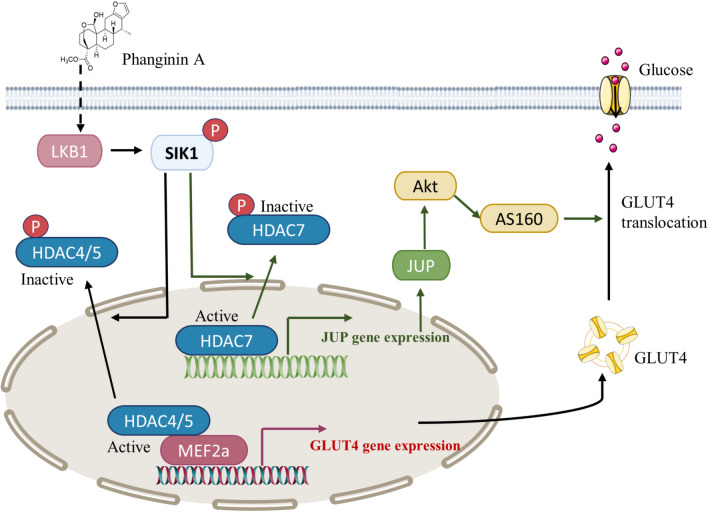
The mechanism by which phanginin A activated SIK1 to promote glucose uptake in skeletal muscle. SIK1 activation leads to the inactivation of HDAC4/5, relieving the inhibition on MEF2a and consequently promoting GLUT4 expression. Additionally, SIK1 activation induces inactivation of HDAC7, resulting in the upregulation of JUP expression and Akt/AS160 signaling pathway, and thus facilitating GLUT4 translocation

In conclusion, this study elucidated a novel mechanism that activation of SIK1 by phanginin A increased glucose uptake of skeletal muscle by both enhancing GLUT4 expression and GLUT4 translocation via inactivating HDAC4/5/7 (Fig. 6). Activation of SIK1 phosphorylated HDAC4/5 to relieve the inhibition on MEF2a and thus promoted GLUT4 expression; SIK1 also phosphorylated HDAC7 to increase JUP expression and PI3K/Akt/AS160 pathway signaling and thus promoting GLUT4 translocation. These findings contribute to a deeper understanding of SIK1-mediated regulation of glucose uptake in skeletal muscle, offering potential insights for the development of therapeutics strategies targeting glucose metabolism.

## Methods

### Animals

C57BL/6 J male mice were purchased from Shanghai Jihui Laboratory Animal Care Co., Ltd. (Shanghai, China) and fed a normal diet (Shilin, Shanghai, China). The mice were housed in a specific, pathogen-free class laboratory under a 12 h light/dark cycle. All animal procedures were approved by the Institutional Animal Care and Utilization Committee (IACUC) (Shanghai, China) of Shanghai Institute of Materia Medica, Chinese Academy of Sciences (2021-08-LY-118).

### Cell culture

C2C12 myoblasts (American Type Culture Collection, ATCC) were maintained in DMEM containing 10% FBS at 37 °C and 5% CO_2_. The confluent cells were differentiated into myotubes by culturing with DMEM containing 2% horse serum (HS) for 5 days.

### Glucose uptake in C2C12 myotubes

C2C12 myotubes were treated with different doses of phanginin A for 24 h and were then incubated in KRHB (130 mM NaCl, 5 mM KCl, 1.3 mM MgSO_4_, 1.3 mM CaCl_2_, 25 mM HEPES, pH 7.4) with or without 100 nM insulin for 30 min; this was followed by incubation with 0.05 mM 2-deoxy-d-glucose and 0.5 μCi/mL 2-deoxy-D-[1, 2-^3^H] glucose at 37 °C for 10 min. The glucose uptake was terminated by three quick washes with ice-cold PBS and followed by addition of 150 μL of 0.1% (vol/vol) Triton-X 100. The radioactivity in cell lysate was determined by liquid scintillation counting (Perkin Elmer Trilu, Massachusetts, USA). The data were normalized against protein concentration, as determined by a Bradford assay.

### RNA isolation and real-time PCR

Total RNA was extracted from C2C12 myotubes or muscle tissues using TRIzol reagent (Cat. 15596018CN, Life Technologies, Carlsbad, CA, USA). cDNA was generated by a Primer Script RT reagent kit (TaKaRa Biotechnology, Dalian, China) and analyzed via quantitative PCR. All of the primer sequences used in this study are included in Table 1. The relative amount of individual mRNA was normalized to GAPDH mRNA.

**Table 1:** The sequences of primers

Gene	Forward sequences (5′-3′)	Reverse sequences (5′-3′)
SIK1	CGCTCAACCCTCCTTGCATA	CGAGGCCAATCTGACTGGAG
SIK2	GCTGCCTTTATGGAGGAAGAGTG	GAGGTCTCCATCATACTGCTGG
SIK3	AACGGCAGCTAGGACAACAGTC	GTGTTGGAGTCCTTGTAGGTGG
GLUT4	GGAACACTGGTCCTAGCTGT	ATCATGCCACCCACAGAGAA
JUP	CTGTGTGCCCTCTGTAAGCA	GAACTGTCCTCGCCTGAGAC
MEF2a	ACACGCATAATGGATGAGAGGAACC	CAACGATATCCGAGTTCGTCCTGCT
PDK4	GTCGAGCATCAAGAAAACCGTCC	GCGGTCAGTAATCCTCAGAGGA
HK2	TGCCACTCCAGACGGTACAGA	TCTCTACGCCCCTTCGCTTG
PDK1	CCACTGAGGAAGATCGACAGAC	AGAGGCGTGATATGGGCAATCC
PKM	CAGAGAAGGTCTTCCTGGCTCA	GCCACATCACTGCCTTCAGCAC
GAPDH	AGGTCGGTGTGAACGGATTTG	TGTAGACCATGTAGTTGAGGTCA

### Western blotting

C2C12 myotubes were pretreated as described, and then the myotubes were homogenized and subjected to western blot analysis as described previously [36]. The membrane proteins were prepared using the Membrane and Cytoplasmic Protein Extraction Kit (P0033, Beyotime, Jiangsu, China) according to the manufacturer’s protocol. The primary antibodies against phospho-Akt (Ser473) (# 9271), phospho-Akt (Thr308) (#9275), pan-Akt (#9272), LKB1 (#3047), GLUT4 (#2213), phospho-AS160 (Thr642) (#8881), phospho-HDAC4/5/7 (Ser426/Ser259/Ser155) (#3443), GAPDH (#2118), β-Actin (#4970) and Na, K-ATPase (#3010) were purchased from Cell Signaling Technology (Danvers, MA, USA). Antibody against phospho-SIK1 (Thr182) (PA5-64610) and SIK1 (ab217809) were from Invitrogen (Carlsbad, CA, USA) and Abcam (Cambridge, MA, USA), respectively. Antibodies against HDAC4 (CY5914), HDAC7 (CY5901) and MEF2a (DY1617) were obtained from Abways (Shanghai, China). Antibodies against HDAC5 (F0948), AS160 (F0418) were obtained from Selleck Chemicals LLC (Houston, TX, USA). The protein marker was purchased from Thermo Fisher Scientific Inc. (Cat.26617, Waltham, MA, USA) and Yeasen Biotechnology (Cat.20350ES90, Shanghai, China). Equal amounts of proteins were separated by SDS-PAGE, transferred to polyvinylidene difluoride membranes (Bio-Rad, Hercules, CA, USA). The blots were blocked in 7.5% milk and then incubated with primary antibodies overnight at 4 °C, followed by 1 h incubation with the horseradish peroxidase-conjugated secondary antibodies at room temperature. The blot bands were visualized via ECL plus chemiluminescence (RPN2236, Amersham, IL, USA) and were quantified via densitometry using Quantity One (Bio-Rad).

### RNA interference

C2C12 myotubes were transfected with selective siRNA using Lipofectamine 3000 RNAiMAX Transfection Reagent (Thermo Fisher Scientific, Waltham, MA, USA) for 48 h for follow-up experiments. The siRNA of SIK and JUP was obtained from Life technologies (Gaithersburg, MD, USA). LKB1 siRNA was provided by GenePharma (Shanghai, China). AMPK siRNA was purchased from Santa Cruz Biotechnology (Santa Cruz, CA, USA). Non-silencing RNA was purchased from Gene-Pharma (Shanghai, China) and used as a negative control.

### Treatment of phanginin A in C57BL/6 J mice

The C57BL/6 J male mice (7–8 weeks old) were stratified divided into two groups according to body weights (n = 8 per group) and orally administered once daily with 100 mg/kg of phanginin A or vehicle (0.25% CMC-Na) for 16 days. On the last day, the mice were anesthetized, gastrocnemius muscles were dissected and stored at − 80 °C for further analysis. The glycogen content in gastrocnemius muscle was measured following the manufacturer’s protocol of using a glycogen content kit purchased from Suzhou Comin Biotechnology (Suzhou, China).

### Statistical analysis

Results are illustrated as mean ± standard error of the mean (SEM) of at least three replicates if not indicated otherwise. Statistical analysis between two groups was determined using the unpaired two-tailed student's t-test. The comparison of multiple groups was carried out using one-way ANOVA followed by Dunnett’s test in GraphPad Prism software. Values of P < 0.05 were considered statistically significant.

## Data Availability

Data will be made available on request.

## Abbreviations

SIK1: Salt-inducible kinase 1
GLUT4: Glucose transporter 4
JUP: Junction plakoglobin
PI3K: Phosphatidylinositol 3-kinase
Akt: Protein kinase B
AMPK: AMP-activated protein kinase
NO: Nitric oxide
ROS: Reactive oxygen species
MEF2: Myocyte enhancer factor 2
GEF: Glucose enhancer factor
HDACs: Histone deacetylases
CaMKII: Calcium/calmodulin dependent kinase II
AS160: Akt substrate of 160 kDa
PDK1: Pyruvate dehydrogenase kinase isozyme 1
PKM: Pyruvate kinase M
HK2: Hexokinase 2
PDK4: Pyruvate dehydrogenase kinase isozyme 4
